# Three novel ABCC5 splice variants in human retina and their role as regulators of ABCC5 gene expression

**DOI:** 10.1186/1471-2199-8-42

**Published:** 2007-05-23

**Authors:** Jelena Stojic, Heidi Stöhr, Bernhard HF Weber

**Affiliations:** 1 Department of Gynaecology and Obstetrics, University of Wuerzburg, Josef-Schneider-Str. 4, 97080 Würzburg, Germany; 2 Institute of Human Genetics, University of Regensburg, Franz-Josef-Strauss-Allee 11, 93053 Regensburg, Germany

## Abstract

**Background:**

The ABCC5 gene encodes an organic anion pump of the ATP-binding cassette (ABC) transporter family, subclass C. The exact physiological function of ABCC5 however is not known. Here, we have isolated three novel ABCC5 splice variants and characterized their role in the regulation of ABCC5 gene expression.

**Results:**

Two additional exons within intron 5 of the ABCC5 gene were identified; one of the exons exhibits alternative donor splice sites. Differential usage of these exons generates three short ABCC5 transcripts named ABCC5_SV1, ABCC5_SV2 and ABCC5_SV3. The variants share the first five exons with the ABCC5 gene but differ in their 3' sequences. ABCC5 and its novel isoforms are abundantly expressed in the human retina. Splice variant ABCC5_SV1 and ABCC5_SV2 contain premature stop codons. While inhibition of nonsense-mediated mRNA decay selectively stabilized ABCC5_SV1 but not ABCC5_SV2, the amount of full length ABCC5 mRNA was simultaneously reduced. A negative regulatory effect on full length ABCC5 expression was also observed when the ABCC5 isoforms were silenced with siRNA duplexes. Finally, we show that the evolutionarily conserved ABCC5_SV2 transcript is translated into a protein abundantly present in endothelial cells of inner retinal blood vessels and along RPE membranes.

**Conclusion:**

Our data suggest that alternative splicing of the ABCC5 gene has functional consequences by modulating ABCC5 gene expression. In addition, at least one ABCC5 splice variant is protein-coding and produces a truncated ABCC5 protein isoform with thus far unknown functional properties in the retina.

## Background

ATP-binding cassette (ABC) transporters are integral membrane proteins which mediate the ATP-dependent translocation of a wide variety of compounds across extra- and intracellular membranes [1]. The substrate diversity ranges from small inorganic ions, amino acids, peptides, sugars, lipids, and anticancer drugs to large proteins. ABC transporters are characterized by a basic modular architecture consisting of two membrane spanning segments and two intracellular nucleotide binding domains with Walker motifs A and B and an ATP-binding cassette signature [1].

Based on protein sequence homology and phylogenetic analyses, the 56 mammalian ABC transporters have been classified into seven subfamilies with the closely related multidrug resistance proteins (MRPs) grouped together in the C branch of ABC proteins (ABCC). ABCC5 (MRP5) is a typical organic anion pump and belongs to the short type of ABCC proteins which differ from the long type by the lack of an N-terminal transmembrane domain [2]. *In vitro *transport studies identified ABCC5 as a cellular export pump for numerous compounds including cGMP [3], nucleoside monophosphate analogs [e. g. [4,5]], heavy metal compounds and fluorochromes [6]. ABCC5-transfected cells were also reported to exhibit resistance to anticancer and antiviral drugs [5,7]. The affinity of ABCC5 to its substrates, however, has generally been low. This suggests that the biological significance of ABCC5 as a mediator of active cGMP efflux, its possible role in drug resistance and ultimately its physiological function is still unknown.

Previous mRNA expression studies showed that the ABCC5 gene is widely transcribed among human tissues with highest levels in heart, brain, skeletal muscle, kidney and testis [6,8]. Multiple mRNA species for various ABCC family members have been described [9,10] including the ABCC5 locus [6]. Sequencing of a single cDNA clone from a human lung cancer cell line identified a splice variant of ABCC5 formed by the alternative usage of a cryptic donor splice site upstream of exon 11 [11]. This so-called short type of multidrug resistance protein homologue (SMRP) translates into an N-terminally truncated version of ABCC5 (946 versus 1437 amino acids) and shows a similar expression pattern as the full length ABCC5 transcript [12]. The physiological relevance of the rare SMRP transcript is not known.

In this study we have characterized three novel isoforms of the ABCC5 gene generated by alternative splicing of newly identified exons within intron 5 of the ABCC5 gene. The various ABCC5 transcripts are abundantly expressed in the human retina but are also present in many other tissues at varying levels. We provide evidence that alternative splicing of the ABCC5 mRNA may provide an elegant mechanism to achieve a tissue-dependant regulation of ABCC5 gene expression.

## Results

### Cloning of three novel ABCC5 isoforms

**Figure 1:**
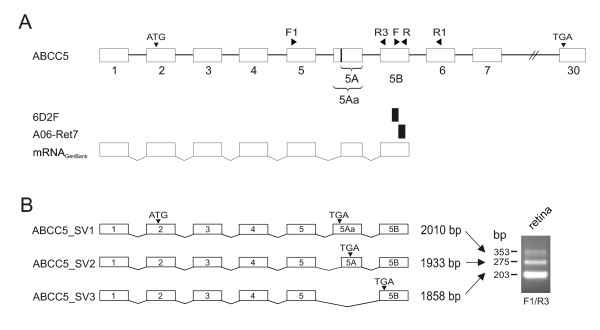
**Cloning of ABCC5 splice variants**. (A) Identification of exon 5A, 5Aa and 5B within intron 5 of the ABCC5 gene. Exons of the ABCC5 gene are shown as open boxes, the retinal cDNA fragments as solid boxes. The mRNA_GenBank _represents a cDNA assembled from three human mRNA sequences deposited in GenBank [BC050744, CR590924 and CR619835]. The locations of primers used to PCR amplify ABCC5 cDNA fragments and the start and stop codon of full length ABCC5 are indicated by solid triangles above. Numbers refer to the respective exon number. The vertical line in exon 5Aa indicates an alternative splice donor site (B) Differential splicing of exons 5A, 5Aa and 5B generates three ABCC5 isoforms in the human retina, termed ABCC5_SV1, ABCC5_SV2 and ABCC5_SV3. RNA from human retina was RT-PCR amplified with primer pair F1/R3.

Large-scale sequencing of a human retinal cDNA library [13] revealed two small cDNA clones, 6D2F (160 bp) and A06-Ret7 (171 bp), located within intron 5 of the ABCC5 gene (Fig. 1A). Subsequent BlastN database queries [14] revealed the retinal sequences to correspond to the 3' end of three overlapping full length cDNA clones isolated from cancer cells and placenta [GenBank: BC050744, CR590924 and CR619835]. After assembly, the human mRNA sequences from GenBank comprise the first five exons of the ABCC5 gene spliced to two alternative exons within intron 5 of the ABCC5 gene, named exon 5A and 5B (Fig. 1A). To further explore the existence of novel ABCC5 splice variants in retina, we performed RT-PCR on human retinal RNA with primers located in exon 5 and exon 5B. Three distinct PCR products of 203, 275 and 353 base pairs (bp) were repeatedly amplified (Fig. 1B). Direct sequencing of the PCR products showed that the two smaller fragments differ by the inclusion of exon 5A. In addition, the usage of an alternative splice donor sequence produces a larger exon 5A (termed exon 5Aa) giving rise to the 353 bp fragment (Fig. 1B). RT-PCR with primers located in exon 5 (F1) and exon 6 (R1) amplified a single 267 bp fragment lacking exon 5A, 5Aa or 5B (data not shown) indicating that the newly identified exonic sequences represent the respective 3'-ends of novel ABCC5 splice variants ABCC5_SV1 (2010 bp) [GenBank:AY754874], ABCC5_SV2 (1933 bp) [GenBank:AY754875] and ABCC5_SV3 (1858 bp) [GenBank:AY754876] (Fig. 1B). This is confirmed by the presence of a potential consensus polyadenylation signal, AATAAA, within 15 bases of the 3' end of exon 5B. Translation of the putative ABCC5_SV1, ABCC5_SV2 and ABCC5_SV3 mRNA sequences revealed stop codons in exon 5Aa, exon 5A and exon 5B, respectively (Fig. 1B). This should lead to putative truncated ABCC5 proteins which share the 197 N-terminal amino acids with the full length ABCC5 transporter and terminate in short distinct C-termini of 15 (ABCC5_SV1), 11 (ABCC5_SV2) and 28 (ABCC5_SV3) amino acids in length.

Nucleotide homology searches using the novel ABCC5 exons as templates showed that the sequence and the flanking splice junctions of exon 5A are highly conserved between mammalian species (e.g. 100% sequence identity to monkey/cow and 98% identity to mouse/dog). Analysis of EST sequences from different species confirmed that exon 5A is commonly spliced to exon 5 whereas each species uses different donor splice sites to join exon 5A to downstream sequences within intron 5 of the ABCC5 gene. Thus, sequences overlapping but not identical to human exon 5B are frequently included in alternative transcripts. The presence of ABCC5 splice variants with additional exons within intron 5 of the ABCC5 gene appears to be a common feature among mammalian species possibly indicating a conserved function of these molecules.

### Expression analysis

**Figure 2:**
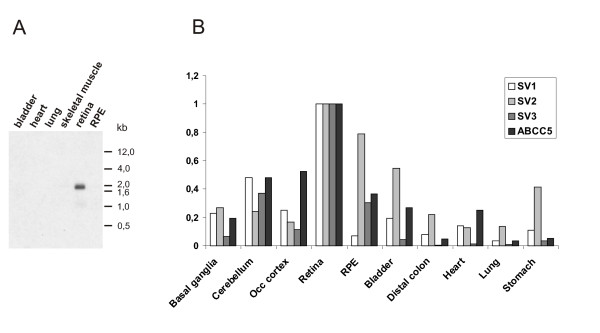
**Expression analysis of ABCC5 splice variants**. (A) Virtual Northern blot probed with an RT-PCR fragment originated from exon 5B. Consequently, the 2 kb signal obtained in retina represents transcripts ABCC5_SV1, ABCC5_SV2 and ABCC5_SV3. (B) qRT-PCR expression analysis of full length ABCC5 and the three splice variants. The expression values for each transcript in the various tissues are indicated by bars in different shades and are given relative to the expression in retina which was set to 100%.

Qualitative RT-PCR analysis revealed varying transcription levels of the novel splice variants in human retina with an increase of expression from ABCC5_SV1 to ABCC5_SV3 (Fig. 1B). The tissue distribution of the novel ABCC5 splice variants was studied by virtual Northern blot analysis with a radiolabeled probe representing exon 5B. A single strong band of approximately 2.0 kb was detected exclusively in retina but not in bladder, heart, lung, skeletal muscle and RPE (Fig. 2A). To further investigate the relative expression of the splice variants, quantitative real-time RT-PCR (qRT-PCR) with primer pairs specific for each of the ABCC5 transcripts was performed with RNA extracted from ten human tissues including samples from three distinct brain areas (Fig. 2B). An overall abundant expression of ABCC5 and the shorter ABCC5 isoforms was observed in human neural retina. In addition, full length ABCC5 is expressed at moderate levels in several tissues including brain, RPE, bladder and heart. A relatively high expression in RPE, bladder and stomach was found for ABCC5_SV2 whereas ABCC5_SV1 and ABCC5_SV3 are only weakly present in nonneuronal tissues. Moderate expression of ABCC5_SV1 and ABCC5_SV3 was detected in cerebellum. Taken together, our data show that each tissue analyzed expresses the various ABCC5 transcripts but at distinct levels suggesting a complex regulation of gene expression.

### Nonsense mediated decay of ABCC5 isoforms

**Figure 3:**
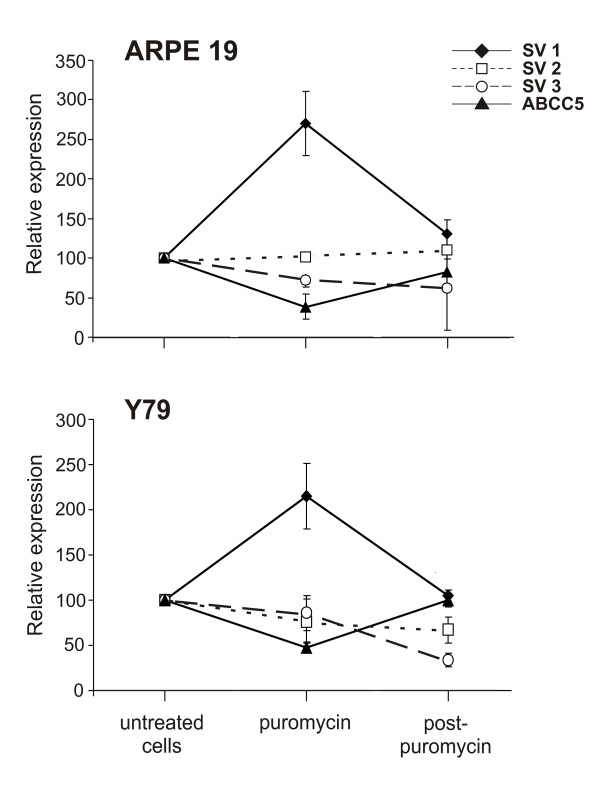
**Inhibition of protein synthesis selectively stabilizes ABCC5_SV1 mRNA**. Total RNA was isolated from untreated ARPE-19 and Y79 cells, from cells exposed to puromycin and from cells grown in regular medium after antibiotic exposure (post-puromycin). The RNAs were subjected to qRT-PCR using primer pairs specific for the ABCC5 splice variants. The expression values for each variant are depicted by different symbols and are given relative to the expression in untreated cells which was set to 100%. Error bars denote standard deviations of reactions performed independently in triplicate.

A stop codon which resides over 50–55 nucleotides upstream from a downstream exon/exon junction is predicted to be recognized by the translational machinery as a premature termination codon (PTC) and thus should be targeted by nonsense mediated mRNA decay (NMD) [15]. Splice variant ABCC5_SV1 contains a stop codon 104 bp upstream of the exon 5Aa/5B splice junction fulfilling the criteria for a PTC. To determine whether the ABCC5_SV1 mRNA is subject to NMD we inactivated the NMD surveillance mechanism by the addition of translation inhibitors puromycin, or alternatively anisomycin, [16] to human cell lines ARPE-19 and Y79 which were shown before to express ABCC5, ABCC5_SV1, ABCC5_SV2 and ABCC5_SV3 (data not shown). By qRT-PCR, puromycin treatment drastically increased the relative expression of ABCC5_SV1 mRNA by almost 3-fold in the ARPE-19 cell line and by more than 2-fold in the Y79 cell line (Fig. 3). Interestingly, in both cell lines the expression of the ABCC5 transcript decreased by a factor of approximately two. In contrast, the relative expression levels of ABCC5_SV2 and ABCC5_SV3 remained unaffected by the addition of the translation inhibitors. The concentration of the mRNAs largely returned to pre-treatment levels after protein synthesis was restored by removing puromycin from the media. Similar to the treatment with puromycin, application of anisomycin lead to a 2- and 4-fold increase of ABCC5_SV1 mRNA in Y79 and ARPE-19 cells, respectively, whereas the levels of the remaining ABCC5 transcript variants were stable (ABCC5_SV2 and ABCC5_SV3) or decreased (ABCC5) (data not shown). Together, these data demonstrate that inhibition of cellular protein synthesis leads to a selective stabilization of the ABCC5_SV1 mRNA indicating that this transcript is regulated by NMD. In addition, elevated levels of ABCC5_SV1 mRNA appear to regulate ABCC5 gene expression without exerting any effects on the remaining short ABCC5 isoforms.

### Silencing of the novel ABCC5 isoforms by RNA interference

**Figure 4:**
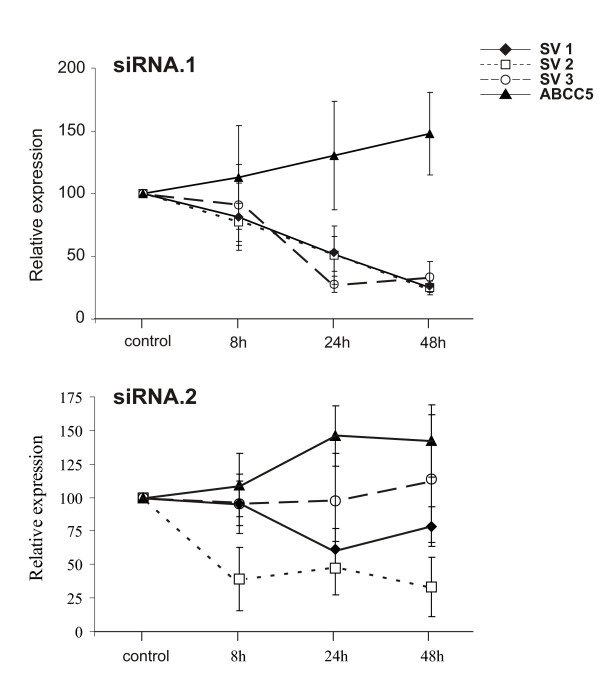
**Suppression of ABCC5 splice variants by RNA interference increases full length ABCC5 mRNA expression**. ARPE-19 cells were transfected with siRNA.1 targeting ABCC5_SV1, ABCC5_SV2 and ABCC5_SV3 or siRNA.2 targeting ABCC5_SV1 and ABCC5_SV2. ARPE-19 transfected with a nonsense siRNA was used as a negative control. Total RNA isolated 8 h, 24 h and 48 h after transfection was used for qRT-PCR analysis. The respective expression values are shown by specific symbols. The expression level of cells transfected with nonsense siRNA was set at 100%. Bar graphs represent standard deviations of reactions performed in triplicate.

To further investigate whether the expression of the various ABCC5 transcripts may be co-regulated, ABCC5_SV1, ABCC5_SV2 and ABCC5_SV3 were silenced by RNA interference. Consequently, treatment of ARPE-19 cells with an siRNA probe targeted to exon 5B (siRNA.1) decreased the expression of ABCC5_SV1, ABCC5_SV2 and ABCC5_SV3 up to 80% while the expression of ABCC5 increased 1.5-fold over time (Fig. 4A). A similar rise in ABCC5 expression was observed when ABCC5_SV1 and ABCC5_SV2 expression was selectively suppressed by siRNA.2 (Fig. 4B). The level of ABCC5_SV3 essentially remained unaffected. siRNA probes to specifically target ABCC5_SV1 without affecting the other two isoforms were designed, but consistently failed to reveal a measurable knock down effect of ABCC5_SV1 expression (data not shown). From our results we conclude that ABCC5 mRNA expression is differentially regulated by the presence of one or more short ABCC5 splice variants, most likely by ABCC5_SV1 and/or ABCC5_SV2.

### Localization of ABCC5_SV2 in the retina

**Figure 5:**
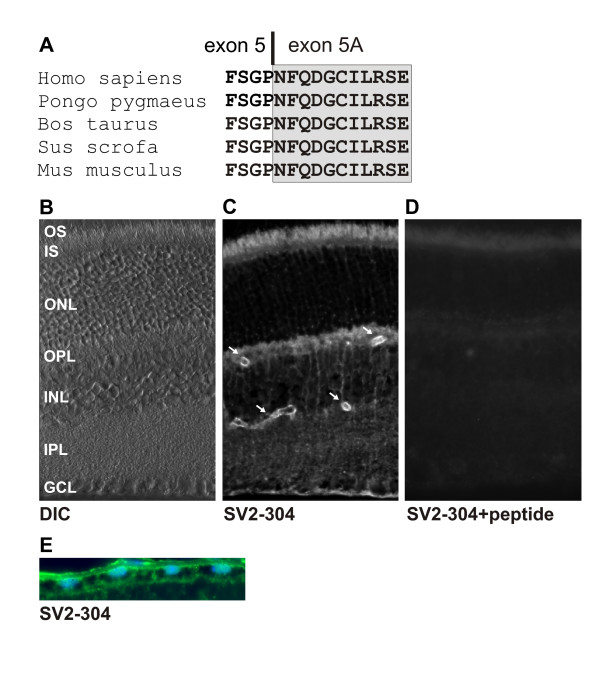
**Localization of the ABCC5_SV2 protein in mouse retina**. (A) Alignment of the immediate C-termini of putative ABCC5_SV2 proteins from several mammalian species reveals 100% amino acid identity. The relative location of the exon 5 – exon 5A boundary is shown by a vertical bar. (B) Differential interference contrast (DIC) image shows the various retinal cell layers: OS, outer segments; IS, inner segments; ONL, outer nuclear layer; OPL, outer plexiform layer; INL, inner nuclear layer; IPL, inner plexiform layer; GCL; ganglion cell layer. (C) ABCC5_SV2 immunolabeling of frozen mouse retina sections with the SV2-304 antibody. Arrows point to labelled endothelial cells of the inner retinal blood vessels. (D) Mouse retinal section stained with SV2-304 antibodies preincubated with an excess of competing GST-ABCC5_SV2 peptide. (E) RPE monolayer labelled with SV2-304 antibodies (green), the nuclei were counterstained with DAPI (blue). The basal side of the RPE is oriented towards the top.

The most C-terminal 11 amino acid peptide specifically encoded by the ABCC5_SV2 isoform appears highly conserved in a number of mammalian species suggesting a functional constraint on this sequence (Fig. 5A). To investigate whether ABCC5_SV2 indeed is expressed as a short ABCC5 protein isoform, we generated polyclonal antibodies (SV2-304) directed against its unique 11 amino acid terminus. Immunofluorescence labeling of frozen mouse retinal sections with purified SV2-304 antibodies repeatedly produced strong staining of endothelial cells of the inner retinal capillaries (Fig. 5B, arrows). Weaker but distinct labelling was also observed in the synapses of the outer plexiform layer and the photoreceptor outer segments. Preadsorption of SV2-304 with GST-ABCC5_SV2 fusion protein completely abolished the immunostaining in the mouse retina (Fig. 5C) indicating that the labelling is specific. In the RPE, a tissue with high ABCC5_SV2 transcription (Fig. 2B), SV2-304 antibodies produced markedly staining of the apical and basolateral surfaces with exclusion of the RPE microvilli (Fig. 5E).

## Discussion

Here we characterize three short ABCC5 splice variants which consist of sequences corresponding to the first 5 exons of the ABCC5 gene but revealing distinct 3' ends. The isoforms are generated by the inclusion of one or two novel exons within intron 5 of the ABCC5 gene and the alternative usage of donor splice sites in one of these exons. In-frame translation of the additional exons introduces stop codons, thus generating unique C-termini. Quantitative real time RT-PCR analysis demonstrates that both, the full length ABCC5 transcript and the shorter ABCC5 splice variants are present at varying levels in a number of tissues while all are predominantly expressed in the human retina. Although ABCC5 mRNA expression has repeatedly been found in neurons of the CNS [6,8], this is the first report of ABCC5 being expressed in the neurosensory retina. ABCC5 can therefore be added to the list of abundant ABC transporters with a function in the eye which for example includes ABCA4, the gene underlying Stargardt's disease [17] and ABCC6, the gene implicated in pseudoxanthoma elasticum [18,19]. So far, a role for ABCC5 in retinal disease has not been determined.

Genome-wide analyses have led to the suggestion that alternative splicing affects the vast majority of genes in many organisms [20,21]. EST-based studies indicated a particularly high level of alternative splicing in neuronal tissues including the retina [22]. The high fraction of splice variants among retinal cDNAs are reflected in numerous reports of alternatively spliced retinal genes [e. g. [13,23]]. Moreover, retina-specific mRNA processing has been reported for genes with a broader tissue distribution [24,25]. The retina is a multilayered tissue composed of a number of distinct cell types that are specialized in their function to transform light energy into electric signals. Alternative splicing is regarded an important mechanism to create protein diversity but also to regulate gene expression [26]. Both processes may well be required to perform and control the complex phototransduction process in the retina and also to establish and maintain the structure and integrity of this unique and highly evolved tissue.

Our results on the functional role of alternatively spliced products of the ABCC5 gene in the retina demonstrate that one isoform, ABCC5_SV1, is a target for NMD. NMD is a post-transcriptional surveillance mechanism in eukaryotic cells used to eliminate newly synthesized mRNAs containing premature termination codons (PTCs) [15,27]. NMD targets which may be generated by mutations or errors in mRNA processing are potentially harmful and need to be cleared. In contrast, alternative splicing to induce NMD is a widely used mechanism for gene regulation, also known as regulated unproductive splicing and translation (RUST) [28]. Our data obtained from NMD inactivation and confirmed by RNA interference show that the expression level of full length ABCC5 transcript is influenced by the presence of alternatively spliced ABCC5 isoforms, in particular ABCC5_SV1. RUST therefore may play a role in ABCC5 gene regulation.

Alternative splicing of genes encoding ABC transporters has previously been reported [9,10,29]. Noticeably, alternative splicing of two evolutionarily conserved PTC-containing exons of the ABCC4 gene produces mRNAs that are degraded by NMD [10]. Regulation of ABCC4 gene expression is thought to be accomplished by facilitating the re-initiation of translation. As a consequence shorter ABCC4 proteins lacking a potentially important amino-terminal linker domain would be generated.

The newly identified ABCC5 splice variants encode putative proteins with isoform-specific C-termini that are predicted to be cytosolic. We have generated an antiserum directed against the conserved ABCC5_SV2 isoform which specifically labels the endothelial cells of blood vessels in the inner mouse retina as well as apical and basolateral surfaces of the RPE. This indicates that in addition to gene regulation, alternative splicing of the ABCC5 gene may be a mechanism to increase protein diversity. A polyclonal antibody directed against the C-terminus of the ABCC5 transporter has been widely used to determine the tissue distribution of ABCC5 in several organs. Among other cell types, this antibody strongly stains capillary endothelial cells in the genitourinary tract [30], in the heart [31] and in the brain [32]. In brain, a contribution of ABC transporters including ABCC5 to the blood-brain barrier is discussed [32]. Similarly, the ABCC5_SV2 isoform could play a role in the inner and outer blood-retinal barrier function possibly by controlling ABCC5 transporter activity.

## Conclusion

Here we show that alternative splicing plays a role in the regulation of ABCC5 gene expression via NMD-related mechanisms. In addition, we present evidence that at least one of the ABCC5 splice variants encodes a functional protein localized to the endothelial cells of the inner retinal blood supply and along RPE membranes. Further studies are needed to determine the precise function of ABCC5 and its regulatory as well as protein-encoding isoforms in the retina. This may also shed light onto a possible contribution of ABCC5 to retinal disease.

## Methods

### cDNA cloning

Oligonucleotide primers F1 (5'-AGA AGA GCT GAA TGA AGT TG-3'), R1 (5'-TTC AAT GCC CAA GTC AGT G-3'), R (5'-AGC CAT CTA ACA GGT CAT C-3') and R3 (5'-TCA GTA AGA TGG CGG TGC AGT-3') were used to RT-PCR amplify ABCC5 cDNA fragments from human retina (Fig. 1A). The PCR products were directly sequenced utilizing the ABI PRISM Ready Reaction Sequencing Kit and the ABI 310 automated sequencer (PerkinElmer Life Sciences GmbH).

### Expression analysis

For virtual Northern blot analysis full-length double stranded cDNA was synthesized from 3 μg of total RNA using the SMART cDNA Library Construction Kit (BD Biosciences Clontech) according to the supplier's instructions. Amplification was performed in 19–22 cycles. The cDNAs were separated electrophoretically and transferred to Hybond N+ membrane. A 362 bp DNA fragment from exon 5B of ABCC5 was obtained by RT-PCR amplification with primer pair F (5'-GAA AGA CCC AGA AGG ATG-3')/R and was [α-^32^P]-dCTP radiolabeled to be used as a probe for filter hybridization at 58°C. The filter was exposed at -80°C for 3 days.

The human total RNA master panel including RNAs from 21 different human tissues was purchased from BD Biosciences. RNA from post-mortem retina and retinal pigment epithelium (RPE) was isolated using the RNeasy Total RNA System Kit (Qiagen). First-strand cDNA was generated from RNA samples by reverse transcription using Superscript II (Invitrogen) and served as a template for subsequent PCR assays. Real-time quantitative RT-PCR (qRT-PCR) was performed as described previously (Krämer et al., 2004). Primer pairs for qRT-PCR analysis and fragment sizes were as follows: ABCC5_SV1 (5'-CAA GAA GAG CTG AAT GAA GT-3' and 5'-ACA GCA CCA AGC AAG TGG TC-3', 147 bp), ABCC5_SV2 (5'-GGC AAG AAG AGC TGA ATG AAG T-3' and 5'-CAG CCA TCC TGA AAA TTT GGT-3', 151 bp), ABCC5_SV3 (5'-GGC AAG AAG AGC TGA ATG AAG T-3'and 5'-CAG TCT CCA AAG GAA GGT GGT-3', 151 bp). Average normalization factors were calculated based on four human housekeeping genes (B2M, TBP, SDHA and HPRT) which displayed a stable expression in all tissues. These factors were then used to determine the relative normalized expression values of the ABCC5 transcript variants. All samples were analyzed in triplicate.

### Inhibition of protein synthesis

The RPE cell line ARPE-19 was cultured in DMEM/Ham's F12 (1:1 mixture) supplemented with 2 mM L-glutamine, 15 mM HEPES, 42 mM NaHCO_3 _and 10% FCS. The retinoblastoma cell line Y79 was grown in DMEM containing 10% FCS. In both cell lines protein synthesis was inhibited by addition of 100 μg/ml puromycin or 100 μg/ml anisomycin (Invitrogen) for 2 hours. The cells were washed with PBS and transferred to medium without antibiotics for another 4 hours. Total RNA was then extracted as described above.

### RNA interference

The siRNA duplexes (HPP grade) were purchased from Qiagen. The siRNA.1 was designed to target sequence 5'-AAT TCA GCG TAG CTA CCT CCA-3' and siRNA.2 to target sequence 5'-AAT CTC TCG CCA AGA GTT CAG-3'. A non-targeting siRNA (5'-AAT TCT CCG AAC GTG TCA CGT-3') was used as a control. Cells were seeded in 6-well plates at a density of 1 × 10^6 ^cells/well 24 hours prior to transfection. Transfections were performed with 2 μg siRNA per well using the TransMessenger Transfection Reagent (Qiagen) following the manufacturer's suggestions. Total RNA isolated from the cells at 8, 24 and 48 hours after transfection was subjected to qRT-PCR.

### Immunohistochemistry

An ABCC5_SV2 antiserum (SV2_304) was generated by immunizing rabbits with a GST-ABCC5_SV2 fusion protein containing the unique 11 C-terminal amino acids. The polyclonal antibodies were affinity-purified using a ()HiTrap NHS-activated sepharose HP column (Amersham Biosciences). Mouse posterior eyecups were immersion-fixed in 4% paraformaldeyde in 0.1 M phosphate buffer (PB [pH 7.4]) for 1 hour and cryoprotected in 0.1 M PB containing 18% sucrose for 4 h. The eyecups were embedded in OCT embedding medium (Tissue-Tek), fast frozen in liquid nitrogen and cryosectioned vertically at 10 μm. Cryosections were blocked with 0.1 M PB containing 0.3% Triton X-100 and 10% goat serum for 30 minutes and labeled for 12 hours with SV2_304 diluted 1:100 in 0.1 M PB, 0.1% Triton X-100 and 2.5% goat serum at room temperature. After washing in 0.1 M PB, the sections were incubated with the secondary antibody goat anti-rabbit IgG conjugated to Alexa 488 (Invitrogen) diluted 1:800 for 1 h. Labeled sections were washed in 0.1 M PB, mounted (Confocal Matrix) and examined under an Axioskop-2 mot plus fluorescence microscope (Zeiss). To evaluate the specificity of the SV2_304 antibody, 100 μl of diluted purified antibody was preadsorbed for 4 hours with 100 μg of GST-ABCC5_SV2 fusion protein immobilized on glutathione sepharose beads. The beads were pelleted at 1000 × g for 3 minutes and the supernatants were used for immunohistochemistry as described above.

## Authors' contributions

JS carried out the DNA/RNA experiments and drafted a first version of the manuscript. HS performed the immunohistochemical analysis and greatly revised the manuscript. BHFW designed and supervised the study and edited the manuscript. All authors read and approved the final version of the manuscript.
